# Ectopic eruption of a permanent mandibular tooth in a miniature horse: case report

**DOI:** 10.1007/s11259-026-11119-1

**Published:** 2026-02-25

**Authors:** Rubens Peres Mendes, Max Santana Gonzaga, Murillo Martinez Matheus, Mauricio José Bittar, Renata Gebara Sampaio Doria, Rodrigo Romero Corrêa

**Affiliations:** 1 Department of Surgery, School of Veterinary Medicine and Animal Science, University of São Paulo, São Paulo, SP Brazil; 2 Self-Employed Veterinarian, BittarVet, Rio Claro, SP Brazil; 3 Department of Veterinary Medicine, Faculty of Animal Science and Food Engineering, University of São Paulo, Pirassununga, SP Brazil

**Keywords:** Ectopic eruption, Extraction, Mandible, Tooth, Equine dentistry

## Abstract

Developmental and eruption abnormalities are common and can result in progressive dental diseases. Ectopic eruption of the affected tooth may be the result of these processes. This study aims to report the occurrence of a permanent mandibular tooth in a horizontal position, with retention of the corresponding deciduous tooth, in a miniature horse. A 2-year-old female miniature horse was admitted to the veterinary hospital with a hard swelling on the left mandibular ramus, which had been growing continuously for three months. After a complete evaluation, it was concluded that the swelling was tooth 307, unerupted, immature, in a horizontal position between teeth 306 and 707. It was decided to extract tooth 307, keeping the corresponding deciduous tooth as a mechanical barrier to food entry. The extraction was performed by lateral alveolotomy, syndesmotomy, and retrograde repulsion. Postoperative alveolar infection progressed with apical contamination of tooth 707, requiring its extraction for clinical resolution of the condition. After 60th postoperative day, adequate alveolar healing, reduction in the depth of the intraoral fistula, and complete closure of the external fistula were observed. The patient was discharged from the hospital and has not shown any new clinical signs since then.

## Introduction

Abnormalities in tooth development and eruption result in various clinical conditions. Developmental changes present during eruption usually trigger secondary conditions that worsen as the eruptive process progresses, making interrelated diseases frequent at the time of patient care (Easley 2006).

The tooth eruption process is described in six anatomical stages, beginning with the preparatory phase, characterized by the opening of the bone crypt, followed by the migration of the tooth toward the oral epithelium, until the onset of clinical eruption, identified by the emergence of the crown in the oral cavity. Subsequently, primary occlusal contact occurs, followed by total occlusal contact, ending with the continuous movements of eruption, making the stages interdependent (Baker 2005). In cases where there are failures in the eruption path, either due to changes in tooth position or insufficient space, dental conditions may arise (Baker 2005; Barakzai 2011; Dixon et al. 2000).

Eruption abnormalities occur at any stage and may be traumatic, infectious, or congenital in origin (Baker 2005; Easley 2006). Trauma related to adjacent tissues during tooth development can induce disorders in the dental follicle (Baker 2005; Dixon and Dacre 2005; Dixon 2011), triggering ectopic eruption (Easley 2006; Yaseen et al. 2011), and occasionally, teeth may not erupt (Edwards 1993).

Animals with disorders in the eruption process of premolars and molars are accompanied by nonspecific signs of dental disease, such as behavioral problems, dysmasesia, and facial asymmetry (Dixon et al. 1999; Edwards 1993). In cases where the clinical condition can only be resolved by extracting the affected tooth, the dental condition, the age of the animal, and the number of teeth affected determine the surgical technique to be used (Menzies & Easley, 2014).

Miniature breeds, because they have the same number and size of teeth as standard breeds, may present abnormalities in the eruption process (Menzies and Easley, 2014). However, although this assumption has traditionally been considered empirical, Clauss et al. (2022) demonstrated that tooth size in smaller horses is not proportional to skull size. Additionally, Heck et al. (2019) showed that miniature horses exhibit cranial characteristics similar to those of young non-miniature horses. Delayed eruption, tooth fractures, and supernumerary teeth have been reported as uncommon findings in miniature horses (Tinsley et al. 2023), however, to date, no cases of ectopic tooth eruption have been described in this breed.

Ectopic eruption is a concept widely used in human dentistry, defined by a deviation from the normal pattern of permanent tooth eruption, characterized by tooth eruption in atypical (ectopic) positions (Yaseen et al. 2011). Thus, this report aims to describe the diagnosis and treatment of an ectopic mandibular tooth in a miniature horse.

## Case presentation

**Fig. 1:**
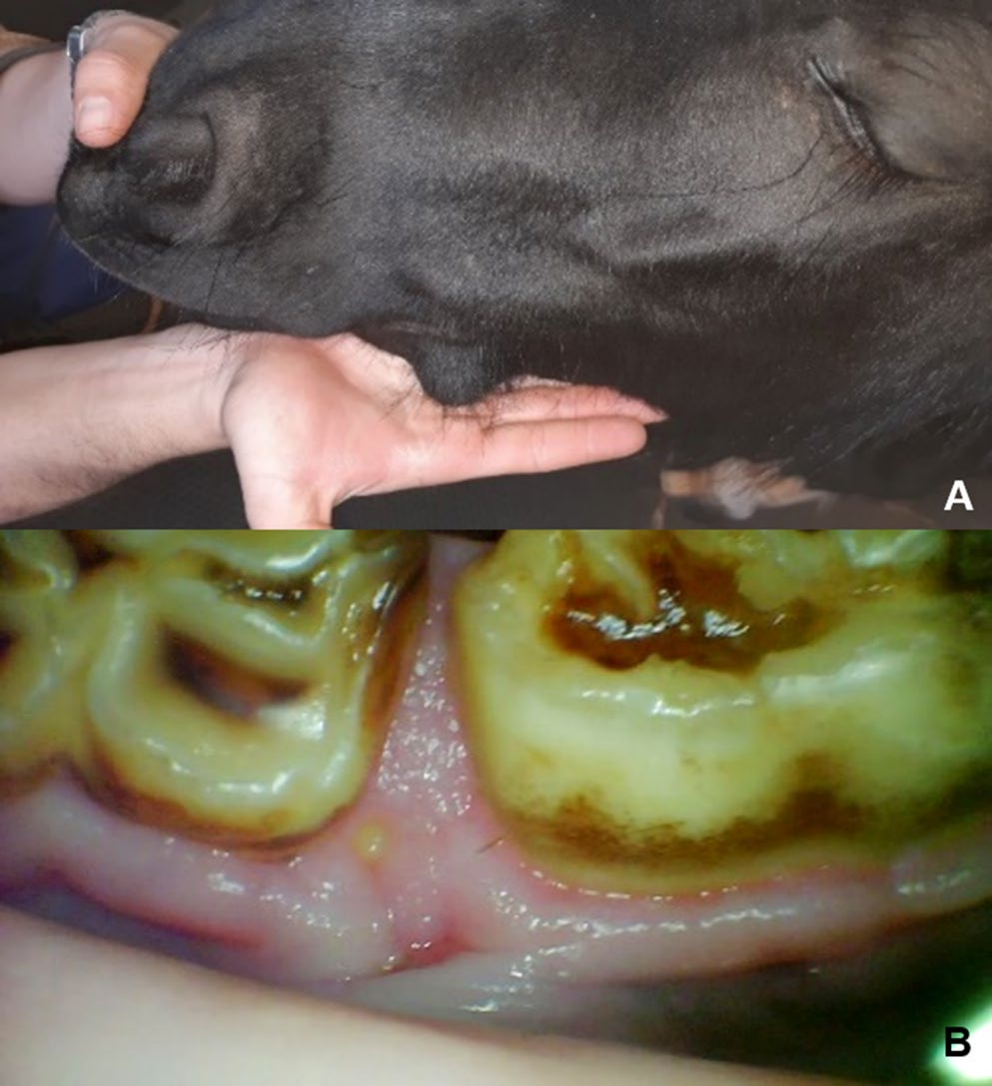
(**A**) Swelling on the lateral side of the left mandibular horizontal branch. (**B**) Diastema between 306 and 707, recorded through oroscopy, in addition to a small periodontal fistula

A 2-year-old female pony weighing 130 kg was admitted to the Clinical Teaching Hospital Unit (UDCH – FZEA/USP) with a history of progressive swelling of the lateral aspect of the left mandibular horizontal ramus over the previous three months. On palpation, it was hard, immobile, and without painful sensitivity (Fig. 1A). Intraoral examination revealed a diastema between teeth Triadan 306 and 707, a periodontal fistula in this position, and a small, firm prominence on the lingual portion of the left mandibular horizontal ramus (Fig. 1B).

**Fig. 2:**
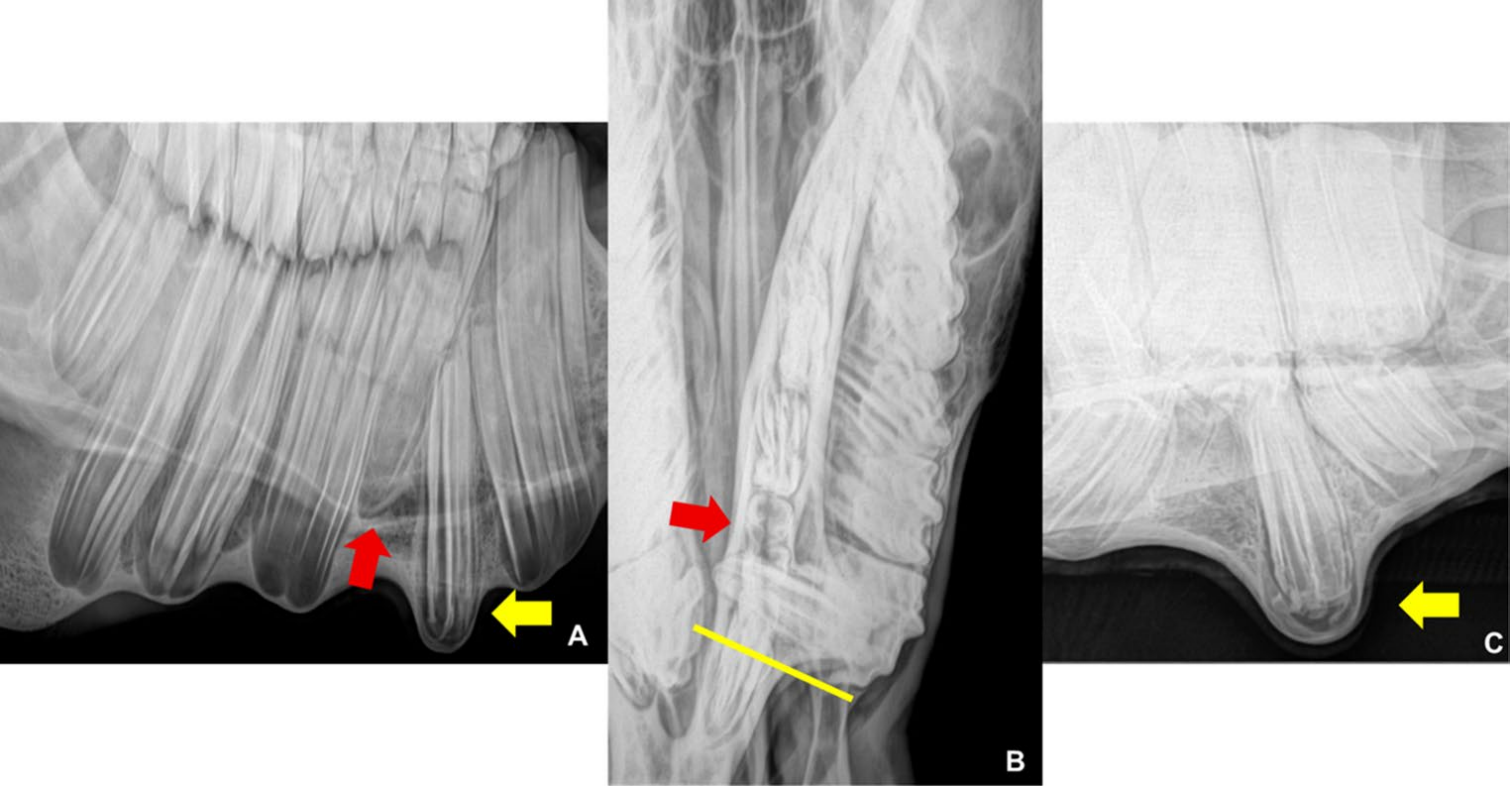
(**A**) - Left ventral–dorsal oblique radiographic projection showing dysplasia and ectopia of tooth 307 (yellow arrow), mild rostral displacement of the reserve crown of tooth 306, and superimposition of tooth 707 over the reserve crown of tooth 407, originating from the right mandibular arcade (red arrow). (**B**) Tooth 307 in horizontal position, with the region corresponding to the clinical crown crossing the medial aspect of the left mandibular ramus, parallel to the yellow line. Tooth 707 retained in normal topography (red arrow) when compared to teeth 306 and 308. (**C**) Left oblique dorsovetral radiographic projection: dysplasia and ectopia of tooth 307 and apical portion “roots” (yellow arrow)

Radiographic examination of the left ventrodorsal oblique projection revealed dysplasia and ectopia of tooth 307, with slight mesial displacement of the reserve crown of tooth 306. The dorsoventral oblique projection shows the apical portion of the tooth more clearly. The dorsoventral offset projection with the mandible displaced to the right showed that tooth 307 was in a horizontal position, with the region corresponding to the clinical crown crossing the medial aspect of the left mandibular ramus (Fig. 2). Tooth 707 was retained in standard topography when compared to teeth 306 and 308.

Based on the clinical and radiographic examination, extraction of teeth 307 and 707 was indicated. Due to the high degree of complexity expected for performing the technique, based on the ectopic position of the tooth, periodontal integrity, and the patient’s size, it was decided to perform a lateral alveolotomy for extraction with the animal under general inhalation anesthesia.

Detomidine hydrochloride (0.01 mg/kg) was used as pre-anesthetic medication for the surgical procedure. Subsequently, ketamine hydrochloride (2.2 mg/kg) combined with diazepam (0.05 mg/kg) was used for patient induction, and isoflurane (1 CAM) was used for maintenance. Regional anesthesia was performed with perineural block of the mandibular nerve, using 10 mL of 2% lidocaine without vasoconstrictor, combined with skin block with the same drug at the incision sites. For the surgical procedure, the animal was positioned in the right lateral recumbency position.

A linear skin incision approximately 4 cm long was made over the mass, parallel to the horizontal branch of the mandible. Next, using Metzenbaum scissors, the subcutaneous tissue was dissected until the bone mass was exposed. Subsequently, the periosteum was elevated using a periosteal elevator.

**Fig. 3:**
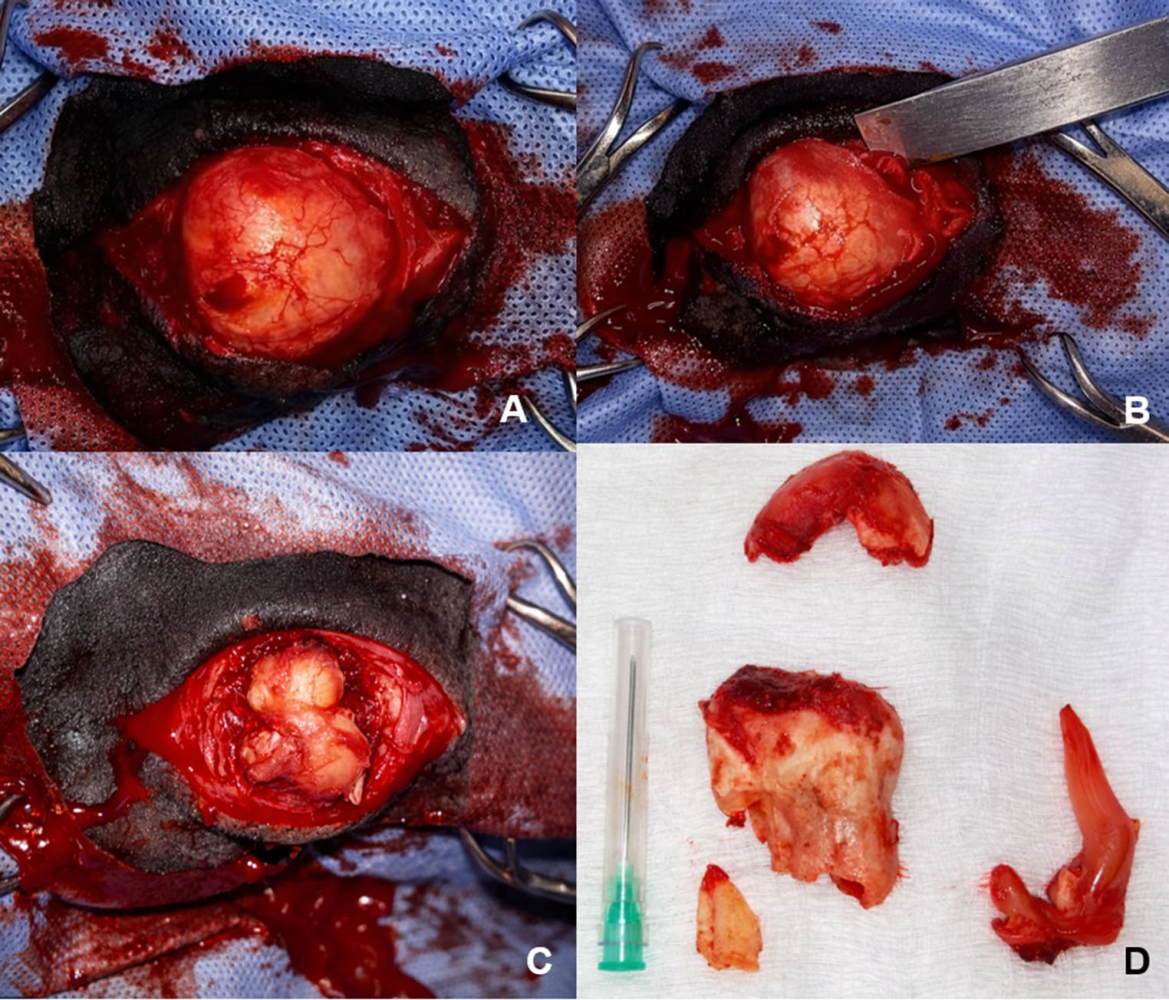
Lateral alveolotomy for extraction. (**A**) Exposure of the lateral plate of the horizontal mandibular ramus. (**B**) Performance of lateral mandibular alveolotomy. (**C**) Exposure of the apex of tooth 307 after alveolotomy. (**D**) Tooth 307 after extraction (red arrow). Fragments of the lateral mandibular cortex (yellow arrow) and pulp segment (black arrow) are also visible

Using an osteotome and surgical hammer, an osteotomy was performed on the lateral aspect of the mandibular ramus until the alveolus of the target tooth was identified. Next, with the aid of bone elevators, an alveolotomy was performed, promoting the removal of the alveolar floor and exposure of the apex of tooth 307 (Fig. 3A and B).

After gaining access to the tooth, an attempt was made to perform retrograde extraction. Using apical levers, syndesmotomy was performed, with rupture of the periodontal ligaments. The exposed apical portion was then grasped with forceps, and controlled rostrocaudal movements were performed to promote tooth dislocation. However, during traction, the reserve crown fractured, making it impossible to continue with the initially proposed surgical technique.

Following the surgical plan, the retrograde repulsion technique was chosen. A 4-mm diameter Steinmann pin was positioned over the dental prominence inserted into the medial aspect of the left mandibular ramus, and through the progressive application of percussive force with a surgical hammer, the dental fragment was detached from the alveolar bone, allowing repulsion to be performed (Fig. 3D).

Postoperative radiographic control confirmed the absence of remaining tooth fragments inside the socket. Tooth 707 was not extracted at the same time, with the aim of using it as a mechanical barrier to prevent food from entering the socket until complete tissue repair.

Postoperatively, treatment was initiated using tetanus serum (10.000 IU), benzathine penicillin (40.000 IU/kg, IM, two applications at 3-day intervals) associated with gentamicin (6.6 mg/kg, IV, SID, for 5 days) and flunixin meglumine (1.1 mg/kg, SID, for 5 days). The alveolar space was filled with dry gauze, and the oral cavity was washed daily with running water. For the surgical wound, daily cleaning with Dakin’s solution was performed, followed by the application of repellent ointment.

On the 15th postoperative day, a new intraoral and radiographic evaluation was performed. Inspection of the oral cavity revealed that the dental alveolus was covered by granulation tissue, with small areas of bone exposure. The communication with the external environment created by the Steinmann pin for repulsion was still present, but with a reduced diameter. Furthermore, the presence of a mandibular fistula was evident on the vestibular and distal sides of tooth 306. Radiographic examination in the left ventrodorsal oblique and dorsoventral *offset* positions with the mandible displaced to the right showed slight filling of the alveolus of tooth 307 with radiopaque bone material.

Thirty days after extraction, the wound was partially covered by epithelial tissue, with a fistula in the cranial portion, which was properly debrided. At the owner’s request, the patient was discharged and care was continued on the property.

**Fig. 4:**
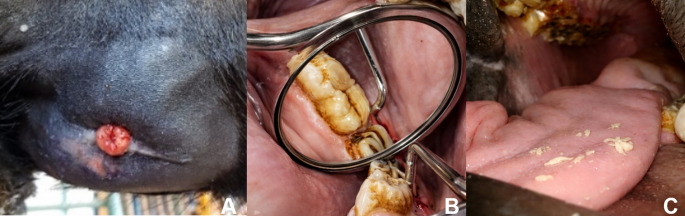
50 days after extraction of tooth 307. (**A**) Increased mandibular volume, with a central scar, exuberant granulation tissue, and diffuse edema.(**B**) Intraoral evaluation using mirror for inspection, showing mesial and vestibular fistulae at tooth 707 and (**C**) Caseous material drained into the oral cavity after retrograde lavage of the fistula

On the 20th day after discharge (50 days after surgery), a new hospital visit was requested. External inspection revealed a new increase in volume at the site where the surgical access had been performed, presenting exuberant granulation tissue at the fistula site (Fig. 4A). Intraoral evaluation revealed persistence of the fistula at the mesial and vestibular margins of tooth 707. Retrograde probing of the fistula was productive, and its irrigation resulted in the drainage of caseous material into the oral cavity (Fig. 4B).

Based on clinical follow-up, intraoral and radiographic examination, it was decided to perform the extraction of tooth 707, in addition to removing the alveolar sequesters that were inside the fistula. The dressing was maintained as described above, using gauze. On the 60th postoperative day, adequate alveolar healing, reduction in the depth of the intraoral fistula, and complete closure of the external fistula were observed. The patient was discharged from the hospital and has not shown any new clinical signs since then. Seventy days after discharge, the owner was contacted by telephone and described complete external healing and reduction in mandibular volume.

## Discussion and conclusion

**Fig. 5:**
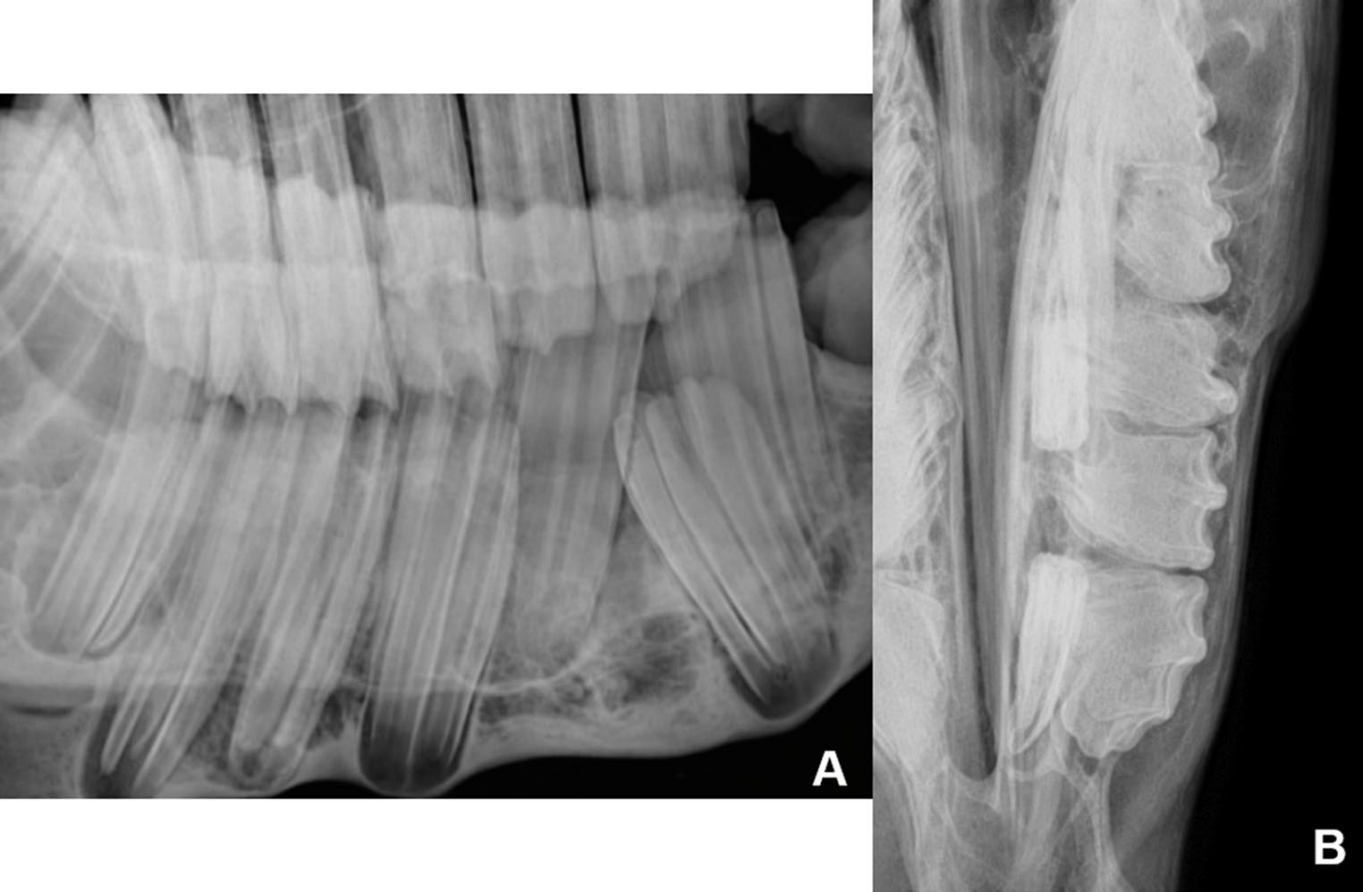
Radiographic examination after extraction of tooth 707, showing filling of the tooth socket of 307 with material of density compatible with bone tissue and remodeling of the mandibular cortex. (**A**) Left ventrodorsal oblique projection, with the mouth open. (**B**) Dorsoventral projection with the mandible displaced to the right

It is known that dental evolution within species can take thousands of years (Dodson and Susarta 2014). This evolutionary process resulted in ponies with small skulls and large teeth, favoring the occurrence of developmental disorders, as observed in the case described. Tinsley et al. (2023) describe the prevalence of several changes involving miniature horses, but the description of ectopia is still considered rare Fig. 5.

Ectopic teeth are malformations caused by the morphogenesis and growth of tissues away from their natural position in the dental arch, which may or may not erupt (Guimarães 1982).

The term ectopic eruption refers to the eruption of permanent teeth occurring outside their normal physiological pattern, resulting in malpositioned teeth or teeth in an abnormal location, generally associated with alterations in the position of the dental germ (Yaseen et al. 2011). In horses, this condition is commonly described as “inappropriate eruption” or “abnormal dental eruption/mal-eruption”, and is defined as a process in which teeth may become rotated or displaced when the dental buds are malpositioned or crowded before, during, or after eruption (Dixon 2021).

Although both terms may be used synonymously, in the present case the term ectopic eruption was considered more appropriate, as no change in the tooth’s topographical location was observed; however, the tooth exhibited an abnormal horizontal orientation of its eruption axis when compared to adjacent teeth. Had surgical intervention not been performed, the tooth would have erupted on the lingual aspect of the oral cavity.

Dixon et al. (1999) included cases of developmental disorders and dental eruption and displacement, rostral (maxillary teeth) and caudal (mandibular teeth) supraversion, diastemas, eruption cysts, supernumerary teeth, and dental displacement. However, the authors in question did not include in their descriptions the non-eruption of horizontally displaced teeth, and the description of these cases in the literature is still lacking (Edwards 1993; Tremaine et al. 2010), making this report rare and important for understanding the condition.

However, premolars are less likely to become displaced during development when compared to molars, since deciduous teeth have approximately the same clinical crown size as the permanent teeth that will replace them (Dixon et al. 1999). It is difficult to pinpoint the cause of the tooth displacement; however, considering the affected tooth, its marked degree of rotation, its unilateral occurrence, and the age of the animal, developmental displacement due to malpositioning of the dental follicle appears more likely.

Edwards (1993) described three cases of permanent tooth retention, two in mandibular teeth and one in a maxillary tooth. As in this case, the author suggested that its occurrence was due to a change in the position of the dental follicle. However, in these two mandibular cases, tooth displacement occurred along the long axis of the tooth at a right angle to its normal eruption line, parallel to the horizontal mandibular ramus, and the teeth never erupted.

The mandibular swelling was the main reason for seeking professional help, with the exception that two of the cases (one maxillary and one mandibular) also presented productive fistulous tracts. None of the cases described by Edwards (1993) presented weight loss or difficulty in the chewing process, corroborating this report.

Although the presence of progressive periodontal disease, probably secondary to diastema, is considered one of the causes of persistent pain and the development of clinical signs, Casey and Tremaine (2010), in a descriptive study conducted on horses with periodontal disease secondary to axial dental changes, did not identify clinical signs compatible with weight loss and dysmasesia. It is believed that the absence of these clinical signs is mainly related to the stoic capacity exhibited by horses, which often mask signs of chronic pain, especially in slowly progressing conditions, as presented in this report.

In this report, the transverse dental positioning to the mandibular horizontal ramus favored the occurrence of mandibular swelling earlier and more abruptly, when compared to the older age of the animals in the cases reported by Edwards (1993). The diagnosis was obtained after radiographic examination (Barakzai 2011; Edwards 1993), given that the only condition found in the intraoral evaluation was diastema. Radiography was essential to establish the cause of the mandibular swelling and was decisive for treatment.

Extraction, being the only viable treatment option, was performed. In the absence of this surgical intervention, dental malocclusion and food impaction with subsequent periodontitis would occur (Dixon et al. 2005a, b), due to the progressive increase in diastema as tooth 307 developed.

Intraoral extraction of teeth with clinical crowns that have not fully erupted is difficult due to poor access to the tooth, obstruction of the eruption path, and the absence of a crown that has erupted sufficiently to be grasped by appropriate instruments (Dixon et al. 2005a, b; Langeneckert et al. 2015; Tremaine et al. 2010). As a result, other extraction techniques may be necessary, such as repulsion with a transcortical and/or retrograde approach (Dixon et al. 2005a, b; Langeneckert et al. 2015; Tremaine and Schumacher 2011; Tremaine et al. 2010).

The lack of intraoral access, due to the size of the animal and the fact that it was an erupted tooth, associated with the risk of iatrogenic fracture of the left mandibular horizontal branch due to the traction force of the forceps, determined the transcortical and retrograde approach for extraction. The lack of dental material to be grasped, due to root fracture after the first extraction attempt, determined the choice of the repulsion technique.

The traumatic nature of repulsion usually causes damage to the alveoli, surrounding teeth, and maxillary and mandibular bones (Coomer et al. 2011; Dixon et al. 2009). The mandibular and alveolar trauma resulted from the osteotomy performed concomitantly with the alveolotomy. Performing the syndesmotomy before repulsion allowed the surgical technique to be performed in a less traumatic manner, resulting in a lower risk of postoperative complications.

Post-surgical complications caused by tooth repulsion include bone and alveolar sequestration, osteomyelitis or localized septic osteitis, as well as orosinus and/or mandibular fistulas (Coomer et al. 2011; Dixon et al. 2009). The absence of granulation tissue filling, noted during the evaluation on the eighth postoperative day, may be suggestive of alveolar bone sequestration, which can be difficult to identify radiographically (Dixon et al. 2009). The occurrence of alveolar sequestration was confirmed when a bone fragment was retirado during routine wound cleaning on the 18th day. Subsequently, there was progressive formation of granulation tissue that filled the entire alveolar space.

It was decided to extract tooth 707 in a second surgical procedure due to the absence of periodontal disease, in an attempt to reduce surgical trauma and avoid overexposure of the alveolar space after extraction of tooth 307. However, postoperative alveolar infection led to contamination of the deciduous tooth, making conservative treatment impossible. After extraction, clinical resolution of the septic condition was achieved, culminating in hospital discharge.

The decision to maintain tooth 707 as a mechanical barrier was based on a clinical risk–benefit assessment, aiming to prevent food accumulation within the alveolus and subsequent bacterial proliferation. However, this approach was associated with postoperative complications and ultimately required a second surgical intervention. The option of simultaneous extraction of both teeth could potentially have reduced the risk of postoperative infection by eliminating the dental barrier and the need for subsequent procedures. Nevertheless, this strategy was initially avoided due to concerns regarding the creation of a large dead space within an odontodependent mandibular bone, which is known to present a higher risk of sequestration and delayed healing. Therefore, the chosen approach represented a cautious balance.

Although developmental abnormalities and eruption disorders are relatively common, the true incidence of developmental displacement involving erupted teeth remains unknown. In the present case, the clinical evolution suggested the need for enhanced postoperative surveillance, as complete clinical resolution had not been achieved at the time of the initial discharge. Furthermore, limited routine handling of the animal by the owner may have contributed to a delay in the recognition of the infectious process associated with the deciduous tooth.

Despite complications, such as those mentioned in the literature, lateral alveolotomy associated with retrograde repulsion was the only option for the extraction of the erupted tooth.

## Data Availability

No datasets were generated or analysed during the current study.
